# A prospective double-blinded randomized study on drug-eluting stent implantation into nitrate-induced maximally dilated vessels in patients with coronary artery disease

**DOI:** 10.1186/s13063-023-07497-5

**Published:** 2023-07-18

**Authors:** Gwang-Seok Yoon, Seong-Huan Choi, Sung Woo Kwon, Sang-Don Park, Seong-Ill Woo

**Affiliations:** Division of Cardiology, Department of Internal Medicine, Inha University College of Medicine, 27, Inhang-Ro, Jung-Gu, Incheon, 22332 Republic of Korea

**Keywords:** Drug-eluting stent, Coronary artery disease, Vasodilation, Nitrates, Randomized controlled trial

## Abstract

**Background:**

Percutaneous coronary intervention (PCI) has been developed using drug-eluting stents (DES); however, stent implantation is associated with concerns of stent thrombosis and target vessel revascularization (TVR). The stent diameter is a critical factor in TVR and clinical events. The nitrate administration in coronary angiography can dilate the reference vessel diameter, enabling accurate vessel size measurement and optimal stent implantation support. This study was designed to evaluate the effect of stent implantation in the maximally dilated coronary artery in patients with coronary artery disease (CAD).

**Methods:**

This prospective double-blinded randomized (1:1) study is designed to compare the efficacy and safety between DES implantation into the nitrate-induced maximally dilated vessels and conventional DES implantation in patients with CAD. A total of 400 patients who underwent PCI with a sirolimus-eluting stent will be enrolled. The primary endpoint is the mean diameter of the deployed stents. Secondary endpoints include cardiac death, myocardial infarction, stent thrombosis, or ischemia-driven TVR 1 year after the procedure.

**Discussion:**

This study will be the first randomized controlled trial to evaluate the effect of DES implantation on nitrate-induced maximally dilated vessels in patients with CAD.

**Trial registration:**

The trial was registered on 18 June 2021 as Effect of Ultimaster Stents Treated to the Most Dilated Coronary Vessels (ClinicalTrials.gov Identifier: NCT04931784).

## Administrative information

**Table:** 

Title	A prospective double-blinded randomized study on drug-eluting stent implantation into nitrate-induced maximally dilated vessels in patients with coronary artery disease
Trial registration	Effect of Ultimaster Stents Treated to the Most Dilated Coronary Vessels (ClinicalTrials.gov Identifier: NCT04931784)
Name and contact information for the trial sponsor	Terumo Korea, Young-Ho Kim Email: YH_Kim@terumo.co.jp)
Funding	This study was funded by the Terumo Corporation, Tokyo, Japan and was supported by an Inha University research grant. They were not involved in the design of the study or the collection, analysis, and interpretation of data and in writing the manuscript

## Background

Drug-eluting stents (DES) contribute to a significant reduction in in-stent restenosis (ISR) and repeat revascularization [1, 2]. However, stent thrombosis and target vessel revascularization (TVR) after DES implantation remain critical concerns [3, 4]. The stent diameter and the post-procedural minimal lumen diameter are major factors in TVR and clinical events; hence, optimal stent implantation is important [5, 6].

Vasospasm or arterial remodeling is common in atherosclerotic coronary artery disease (CAD) cases [7, 8]. Nitrates are known to induce pharmacological vasodilatory effects through vascular smooth muscle relaxation. CAD affects the response of non-diseased proximal coronary segments to nitrate [9]. In particular, nitrate administration in coronary angiography can dilate the diameter of the reference vessel in CAD to measure the exact vessel size [10]. Many experts have recommended routine nitrate administration in coronary angiography procedures [9, 11].

Therefore, we assumed that there will be difference in minimal lumen diameter after DES implantation between patients with and without use of intracoronary nitrate. Accordingly, this prospective double-blinded randomized study is designed to compare the efficacy and safety between DES implantation in nitrate-induced maximally dilated vessels and conventional DES implantation in patients with CAD.

## Methods

The study protocol is reported in accordance with the SPIRIT statement for randomized trials [12]. The Institutional Review Board of Inha University Hospital has approved the study (INHAUH 2021–02-033).

### Study design

**Fig. 1:**
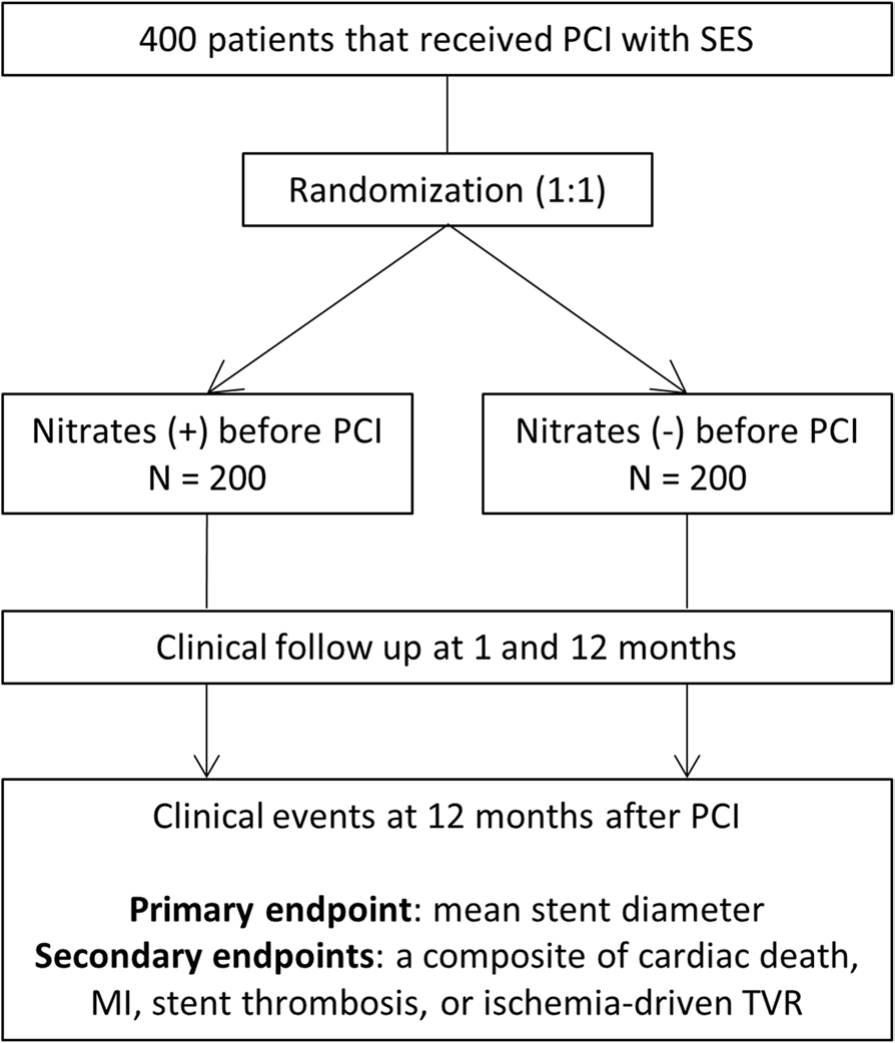
A flow-chart for the study. MI, myocardial infarction; PCI, percutaneous coronary intervention; SES, sirolimus-eluting stent; TVR, target vessel revascularization

A prospective randomized double blinded trial has been designed to evaluate the effect of sirolimus-eluting stent (Ultimaster®, Terumo Corporation, Tokyo, Japan) implantation into the maximally dilated coronary artery. The trial protocol has been registered at http://www.clinicaltrials.gov (NCT04931784), and a brief flowchart of the entire study is presented in Fig. 1.

### Endpoints

The primary endpoint of this study is the mean stent diameter selected at the time of stent implantation. Secondary endpoints include a composite of cardiac death, myocardial infarction, stent thrombosis, or ischemia-driven TVR 1 year after the procedure.

### Patient population

Patients diagnosed with stable angina, acute coronary syndrome, or documented ischemia because of significant lesions will be included. Patients with at least one coronary lesion exhibiting more than 50% diameter stenosis via visual estimation and the reference vessel diameter of 2.25–3.5 mm will be eligible. Patients with (1) hemodynamic compromise within 24 h of the procedure or cardiogenic shock, (2) life expectancy < 1 year, and (3) intolerance to the study drug (nitrates) will be excluded. The inclusion and exclusion criteria are listed in Table 1.

**Table 1:** Inclusion and exclusion criteria

Inclusion criteria
1. Age ≥ 18 years
2. Coronary artery disease with stable or acute coronary syndrome or documented ischemia because of significant lesions
3. Significant coronary artery stenosis (> 50% via visual estimate) considered for coronary revascularization with stent implantation
4. Reference vessel diameter of 2.25–3.5 mm via visual estimation of the surgeon assessment
5. Patients with signed informed consent
Exclusion criteria
1. Contraindication to nitrates
2. Hemodynamic compromise within 24 h before the procedure or cardiogenic shock
3. Life expectancy < 1 year
4. Pregnant women or women with potential childbearing

Patients meeting all inclusion criteria and exhibiting none of the exclusion criteria will be asked to provide a written informed consent after the diagnostic angiogram, required by the Institutional Review Board in accordance with the Declaration of Helsinki.

### Randomization and interventions

A random number table will be generated using the SAS software and independently managed at the Inha University Hospital Cardiovascular Center. Study participants will be randomly assigned in a 1:1 ratio to a group that will receive intra-coronary nitrate before percutaneous coronary intervention (PCI) or a group that will not by the un-blinded coordinator. The result of random grouping (nitrate group or control group) will be placed inside sealed box.

### Blinding

Including interventionist and patients will be blinded to treatment allocation. Injection syringe of nitrate and normal saline (control group) are identical. Unblinded coordinator will make and give the injection syringe to interventionist. This process is conducted and documented according to the respective standard operating procedure. There is no anticipated harm and compensation for trial participation.

### Unblinding

In emergency conditions, up to the decision of the investigator, the blind of the participant is broken, and the allocation codes in the sealed data system are revealed by unblended coordinator.

### Index PCI

Before the procedure, a loading dose of aspirin (300 mg) and P2Y12 inhibitor (600 mg of clopidogrel, 180 mg of ticagrelor, or 60 mg of prasugrel) will be administered to all patients if not used at least 12 h before the PCI procedure. Unfractionated heparin will be used during the procedure to maintain an activated clotting time of > 250 s. An additional dose of 3000 IU of heparin will be added if the procedure extends beyond 1 h. Administration of glycoprotein IIb/IIIa inhibitors will be left to the physician’s discretion. Balloon angioplasty and stent implantation will be performed according to the standard techniques. Intracoronary nitrate will be administered during PCI, typically as a 200–300 mcg bolus. The stent diameter in the nitrate group will be selected via visual estimation or imaging study after the intracoronary nitrate administration, while that in the conventional group will be selected via visual estimation or imaging study without the administration of intracoronary nitrate. The goal of the procedure is to achieve the optimal angiographic efficacy of PCI in selected target lesion sites while minimizing the risk of procedure-related complications. A full range of commercially available guiding catheters, balloon catheters, and guidewires will be readily available. Direct stenting and adjuvant post-dilation will be allowed.

### Post-PCI medication

After PCI, all patients will receive a 100 mg daily dose of aspirin and a P2Y12 inhibitor (75 mg of clopidogrel, 180 mg of ticagrelor, or 10 mg of prasugrel) for at least 6 months. Antiplatelet therapy beyond 6 months will be at the discretion of the treating physician, according to the prevailing guidelines [13].

### Quantitative coronary angiography

Quantitative coronary angiography analysis will be performed using a quantitative coronary angiographic system (CASS system, Pie Medical Instruments, Maastricht, The Netherlands) before and after stent implantation by individuals blinded to the patients’ treatment assignments. The diameters of the reference vessel (the average of the proximal and distal reference lumen diameters), minimal luminal diameter, and percent diameter stenosis (%) will be measured using the guiding catheter for magnification calibration, before and after stenting from diastolic frames, in a single-matched view showing the smallest minimal luminal diameter. All quantitative angiographic measurements will be obtained within the stented segment and the entire segment, including the stent and its 5-mm edge regions.

### Follow-up

The clinical follow-up will be performed with visits until 12 months after hospital discharge. By unblinded coordinator, data will be collected including angina class and major adverse ischemic, neurologic, and bleeding events, including rehospitalization, recatheterization, and adverse events or serious adverse events. All information about electrocardiograms and cardiac enzymes, such as creatine phosphokinase, creatine kinase-MB, and troponin, will be collected through electronic medical record by unblinded coordinator. In case of patients with follow-up loss, data will be corrected over the phone.

### Statistical considerations

#### Sample size calculation

We hypothesized that the stent diameter of maximally dilated coronary arteries using nitrates will be larger than those of the coronary artery of patients who underwent PCI using the conventional method. We assumed an overall increase of 8% in the diameter with the use of nitrates like in previous studies [14]. Assuming a 1% dropout rate, an estimated sample size of 400 patients (200 for each group) is required to achieve 80% power for the superiority test and a two-sided *α* = 0.05. Sample size was calculated with PASS sample size calculator. A statistical package (SPSS16.0) will be used for analysis.

#### Statistical analyses

All data will be presented as percentages or means ± standard deviations. Comparisons of the baseline data using the chi-square test or Fisher exact test (categorical variables) and Student’s *t*-test or Mann–Whitney test (continuous variables) will be performed as appropriate. The Kaplan–Meier method will be used to estimate the cumulative event rate, and data will be compared with the log-rank test. All comparisons will be performed according to the intention-to-treat allocations. *P* values < 0.05 will be considered statistically significant. Major subgroup analyses of primary and major secondary endpoints will be performed. All statistical analyses will be performed using IBM SPSS Statistics for Windows, version 26.0 (IBM Corp., Chicago, Illinois).

### Trial organization

#### Executive committee

The executive committee will comprise the study chairperson and principal investigators of the investigating centers. This committee will approve the final trial design and protocol issued to the data and safety monitoring board (DSMB) and clinical sites. Additionally, this committee will be responsible for reviewing the final results, determining the methods of presentation and publication, and selecting secondary projects and publications by members of the steering committee.

##### DSMB

The DSMB, comprising general and interventional cardiologists, will function in accordance with the applicable regulatory guidelines. Board members are independent and will not participate in the trial. The DSMB committee will review the safety data from this study and make recommendations based on the safety analyses of the unanticipated device effects, serious adverse events, protocol deviation, device failures, and 30-day follow-up reports. The frequency of DSMB meetings will be determined prior to the commencement of the study. Additionally, the DSMB can call meetings at times when it suspects that safety is an issue.

All cumulative safety data will be reported to the DSMB and reviewed as an ongoing process throughout the enrollment and follow-up periods to ensure patient safety. Every effort will be made to allow the DSMB to conduct an unbiased review of patient safety information. All DSMB reports will be made available to the appropriate agencies upon request; otherwise, they will remain strictly confidential.

#### Clinical event adjudication committee (CEAC)

The CEAC comprises interventional and non-interventional cardiologists who are not participants in the study. The CEAC is charged with specific criteria to be developed for the clinical event and endpoint categorizations in the study based on protocol. At the outset of the trial, the CEAC will establish explicit rules outlining the minimum amount of dates required and the algorithm to be followed to classify a clinical event. All members of the CEAC will be blinded to the primary trial results.

The CEAC will regularly review and adjudicate all clinical events in which the required minimum data will be available. The committee will also review and rule out each death throughout the trial.

#### Protocol amendments

The sponsor and the principal investigators are allowed to amend the protocol or to provide suggestions for a protocol amendment. Substantial amendments are only implemented after approval of the competent ethics committee. All non-substantial amendments are communicated as soon as possible or within the competent ethics committee, respectively.

#### Dissemination plans

The results of this research will be disseminated in its whole in international journals. Regardless of study result, both positive and negative findings will be reported.

#### Data sharing

The datasets analyzed during the current study and statistical code are available from the corresponding author on reasonable request, as is the full protocol.

#### Plans for collection, laboratory evaluation, and storage of biological specimens for genetic or molecular analysis in this trial/future use

There will be no biological specimens collected.

### Trial status

The protocol version is number 3.0, dated 5 October 2021. Recruitment has started in October 2021 and is predicted to end in July 2025.

## Discussion

A large prospective randomized study is designed to evaluate the effect of stent implantation on the most dilated coronary arteries using intracoronary nitrates for the treatment of patients with CAD.

Nitrates increase coronary blood flow in the epicardial coronary arteries by vasodilation, imparting an important effect on the coronary artery diameters, especially on the size of non-stenotic segments [14–16]. Spontaneous decreases (of 20%) in luminal diameter have been reported during the course of coronary arteriography in normal humans. A significant decrease in the stenotic segment diameter has been observed with a decrease in the normal adjacent lumen [17]. The effects of nitrates on coronary circulation include coronary artery vasodilation, prevention or reversal of large and small coronary artery vasoconstriction or spasm, coronary stenosis dilation, coronary collateral dilation, and disordered endothelial function improvement [18]. The benefits of nitroglycerin are numerous, from accurate diagnostic angiogram interpretation to the PCI support in simple or complex lesion types. There are opinions that nitroglycerin should be administered in all diagnostic cardiac catheterizations, as well as before, during, and after interventions, unless absolutely contraindicated in extremely rare circumstances [11].

Although numerous studies have evaluated nitrate-induced coronary artery dilation, no studies have reported the effect in patients who underwent PCI. Previous studies have found that intracoronary nitrates increase the coronary artery diameter to 11–17% compared with the baseline diameter using coronary angiography [9, 19]. No trials have so far provided data on whether the coronary artery relaxation induced by nitrates will increase the caliber of vessels, thereby increasing the diameter of the implanted stent and improving clinical outcomes.

Among patients requiring coronary stent implantation, the use of IVUS-guided everolimus-eluting stent implantation, compared with angiography-guided stent implantation, results in a significantly lower rate of the composite of major adverse cardiac events after 1 year post-implantation [20, 21]. These differences are primarily due to the lower risk of target lesion revascularization. The clinical benefit of IVUS-guided DES implantation may be attributed to its larger minimal lumen diameter. The primary contributing factor for the prevention of restenosis after DES implantation is believed to be a larger post-procedural minimal lumen diameter.

Therefore, we designed a clinical study involving stent implantation following intracoronary nitrate administration compared with the conventional stent implantation. We expect that the stent diameter of the most dilated coronary arteries using nitrate will be larger than that of the coronary arteries using conventional PCI. The study may also shed light on the efficacy and safety of stent implantation followed by the intracoronary nitrate administration for the CAD treatment.

In conclusion, this study is the first randomized controlled trial designed to evaluate the effect of DES implantation followed by the administration of intracoronary nitrates for the treatment of CAD. We expect to demonstrate the efficacy and safety of DES implantation in nitrate-induced maximally dilated vessels in patients with CAD.

## Data Availability

The datasets will be available from the corresponding author.
